# Risk of tumour seeding in patients with liver lesions undergoing biopsy with or without concurrent ablation: meta-analysis

**DOI:** 10.1093/bjsopen/zrae050

**Published:** 2024-05-15

**Authors:** Jeremy E Maducolil, Stephanie Girgis, Mohammad A Mustafa, Jayden Gittens, Matthew Fok, Sunanda Mahapatra, Dale Vimalachandran, Robert Jones

**Affiliations:** Department of Surgery, Whiston Hospital, Prescot, UK; School of Medicine, University of Liverpool, Liverpool, UK; School of Medicine, University of Liverpool, Liverpool, UK; School of Medicine, University of Liverpool, Liverpool, UK; Institute of Systems, Molecular and Integrative Biology, University of Liverpool, Liverpool, UK; Institute of Systems, Molecular and Integrative Biology, University of Liverpool, Liverpool, UK; Department of Colorectal Surgery, Countess of Chester NHS Foundation Trust, Chester, UK; Department of Colorectal Surgery, Countess of Chester NHS Foundation Trust, Chester, UK; Institute of Systems, Molecular and Integrative Biology, University of Liverpool, Liverpool, UK; Department of Colorectal Surgery, Countess of Chester NHS Foundation Trust, Chester, UK; Institute of Systems, Molecular and Integrative Biology, University of Liverpool, Liverpool, UK; Department of Surgery, Liverpool University Hospitals NHS Foundation Trust, Liverpool, UK

## Introduction

Liver biopsies are fundamental for research purposes to plan personalized therapies^1^. Needle tract seeding is often raised as a concern during liver biopsies^2^. It occurs during withdrawal of a needle, where malignant cells get displaced along the needle tract. The risk of seeding associated with liver biopsies remains uncertain. In a meta-analysis conducted in 2008, the overall incidence of seeding was 2.7%, ranging from 1.5% to 5.8%^3^. Biopsies at that time were performed using older techniques, such as non-coaxial direct access biopsy, and with small sample sizes. Since then, studies have been performed using newer techniques, such as coaxial percutaneous liver biopsy^2,4–9^. Coaxial biopsy allows multiple samples to be taken through the same tract, whereas non-coaxial techniques require multiple insertions for each tissue sample^4^. Due to concerns regarding needle tract seeding, biopsy with prophylactic ablation of the needle tract has been performed^2,5,10–12^.

The aim of this meta-analysis was to evaluate the risk of needle tract seeding in patients with liver lesions undergoing biopsy with or without concurrent ablation.

## Methods

The review protocol was registered in PROSPERO, the international prospective register of systematic reviews (CRD42022352550), and the systematic review was conducted in accordance with the PRISMA statement^13^.

### Search strategy

A comprehensive search strategy (last updated on 11 November 2023) was developed to search PubMed, Scopus, and Cochrane databases and trial registries. Additional studies were manually searched for by screening the reference lists of the included studies.

### Eligibility criteria

Studies with adult patients (greater than 18 years old) who underwent lesional liver biopsy with or without concurrent ablation were included in this review. Studies published over the last 21 years were included to keep the biopsy and ablation techniques relevant to modern clinical practice. Only studies reported in English were included in this review. Studies with less than or equal to 10 patients were excluded. Conference abstracts, systematic reviews, meta-analyses, and case reports were excluded.

### Statistical analysis

The metafor statistical package in R (version 4.2.3) was used to perform a random-effects meta-analysis of proportions to derive pooled estimates and corresponding 95% confidence intervals^14^. (See the *Supplementary material* for details regarding data extraction and methodological assessment.)

## Results

### Literature search 

**Fig. 1:**
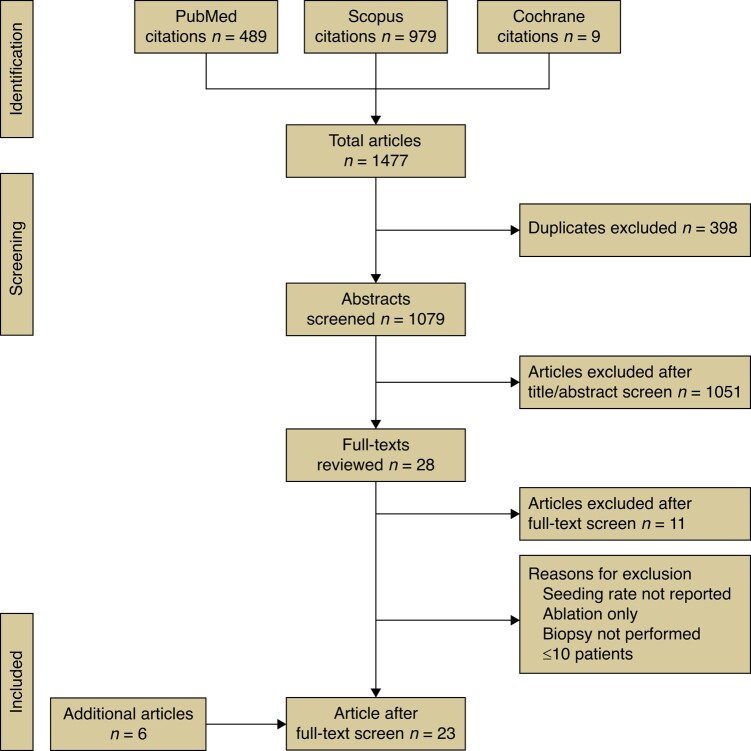
PRISMA flow chart.

After duplicates were excluded, a total of 1079 studies were included. After screening the titles and abstracts, the full texts of 28 studies were reviewed for eligibility. Manual searching of the reference lists identified six additional studies. In total, 23 studies met the inclusion criteria and were included in this meta-analysis. The process is shown in *Fig. 1*.

### Study characteristics

A total of 23 studies were included, representing 8857 patients. The majority of the studies were retrospective studies (19 of 23). Studies were published between 2003 and 2022. Of the patients, 7975 (90.0%) underwent biopsy and 882 (10.0%) underwent biopsy with concurrent ablation. Reasons for performing biopsies were not clearly reported in the included papers. A summary of study characteristics is shown in *Table S1*.

### Needle tract seeding

**Fig. 2:**
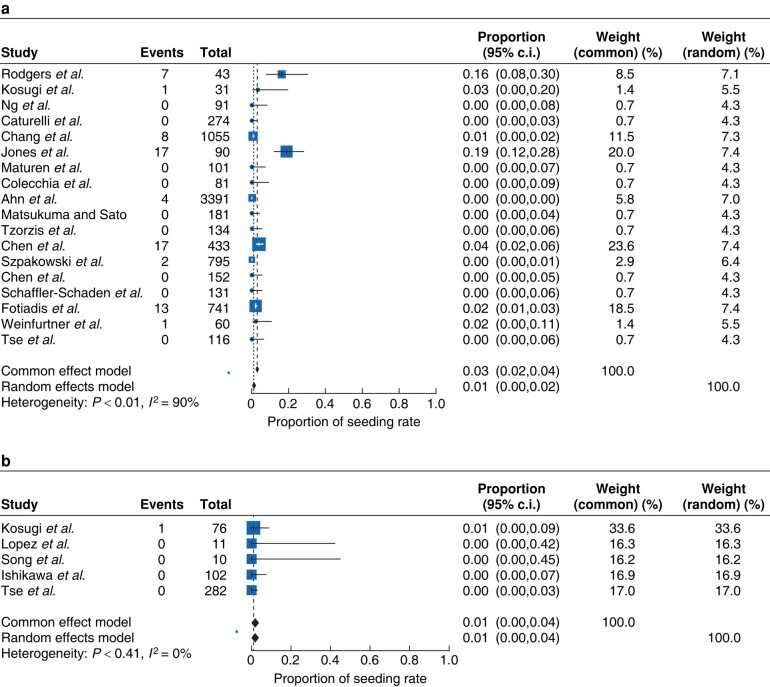
Forest plots **a** Forest plot demonstrating the proportion of seeding rate in patients who underwent biopsy. **b** Forest plot demonstrating the proportion of seeding rate in patients who underwent biopsy with concurrent ablation.

A total of 18 studies were included in the analysis of patients who underwent biopsy. The pooled estimate of the rate of seeding was 0.01 (95% c.i. 0.00 to 0.02; *Fig. 2a*). A single study (Jones *et al.*^15^) stood out as an outlier in the proportional meta-analysis (proportion 0.19 (95% c.i. 0.12 to 0.28)), so a separate proportional meta-analysis without this study was performed (*Fig. S1*). For the random-effects model, the pooled estimate of the rate of seeding was also 0.01 (95% c.i. 0.00 to 0.02), i.e. no different, when the outlier was removed. A total of five studies were included in the analysis of patients who underwent biopsy with concurrent ablation^2,5,10–12^. The pooled estimate of the rate of seeding was 0.01 (95% c.i. 0.01 to 0.04; *Fig. 2b*).

To determine whether the estimated rate of seeding differs in primary liver cancer compared with secondary liver cancer a sub-analysis was performed on the data (*Fig. S2a*,*b*). There were seven studies that reported rates for secondary liver cancer; however, only five were included in the analysis. The pooled estimate of the rate of seeding for primary liver cancer was 0.01 (95% c.i. 0.00 to 0.02) and the pooled estimate of the rate of seeding for secondary liver cancer was 0.03 (95% c.i. 0.01 to 0.09), which was low, but slightly higher than that for primary liver cancer.

### Local recurrence

None of the included studies clearly reported rates of local recurrence in their cohorts.

### Risk-of-bias assessment

All of the included studies underwent risk-of-bias assessment using the Newcastle–Ottawa scale. The results are shown in *Table S2*.

## Discussion

This is the first meta-analysis to evaluate the risk of needle tract seeding in patients with liver lesions undergoing biopsy with or without concurrent ablation. The results show that the risk of seeding was as low as 1% in patients undergoing biopsy with or without concurrent ablation. Thus, is dissemination secondary to percutaneous biopsy less relevant in terms of prognosis than previously thought?

Tissue analysis after biopsy or resection could help to accurately define lesions and tumour grades and could help to develop targeted therapies, providing a gateway to personalized medicine. Biopsy is seldom undertaken due to the perceived risk of adverse events. A method proposed to reduce the risk of seeding is concurrent ablation, which involves ablating the needle tract after the biopsy is taken. There is no standardized protocol and both biopsy with concurrent ablation and biopsy with ablation during a later session have been performed; studies that performed biopsy with concurrent ablation had lower seeding rates compared with studies that performed biopsy and ablation during separate sessions^2,5,10–12,16,17^. Newer techniques, such as coaxial biopsy, had lower seeding rates compared with non-coaxial biopsy techniques^2,4–9^. Coaxial liver biopsies involve inserting a large outer sheath needle into the tissue via which multiple samples can be taken using a smaller needle^4^. Coaxial biopsies also allow for more tissue sampling with fewer complications and could play a vital role in personalized medicine where larger cores are required^4^. Local recurrence rates were not clearly reported in the included papers and were mostly used interchangeably with seeding rates. This may be due to a lack of clear definitions in the literature.

The limitations of this meta-analysis were the relatively small sample size of patients who underwent biopsy with concurrent ablation, the inclusion of all tumour types, and the small number of studies assessing secondary liver cancer. Most of the data came from retrospective studies. Furthermore, the lack of an accepted definition of needle track seeding limits the ability of a meta-analysis to accurately determine the incidence and the impact of this rare event.

These results show a relatively low risk of needle tract seeding in both groups of patients. This can help inform clinicians and multidisciplinary teams when considering the role of liver biopsy and help patients weigh up the risks and benefits of the procedure.

## Supplementary Material

zrae050_Supplementary_Data

## Data Availability

Data are available on request.
