# Supplementary material for: Risk of tumour seeding in patients with liver lesions undergoing biopsy with or without concurrent ablation: meta-analysis
Source: BJS Open. 2024 May 15;8(3):zrae050. doi: 10.1093/bjsopen/zrae050 (PMC11094471; doi:10.1093/bjsopen/zrae050)
Supplement: zrae050_Supplementary_Data [file zrae050_supplementary_data.docx]

**Risk of tumour seeding in patients with liver lesions undergoing biopsy with or without concurrent ablation: meta-analysis**

Jeremy E. Maducolil^1^, Stephanie Girgis^2^, Mohammad A. Mustafa^2^, Jayden Gittens^2,3^, Matthew Fok^3,4^, Sunanda Mahapatra^4^, Dale Vimalachandran^3,4^, Robert Jones^3,5^

1. Whiston Hospital, Warrington Road, Prescot, Merseyside, L35 5DR, UK
2. School of Medicine, University of Liverpool, Liverpool, L69 3GE, UK
3. Institute of Systems, Molecular and Integrative Biology, University of Liverpool, Liverpool, L69 7BE, UK
4. Department of Colorectal Surgery, Countess of Chester NHS Foundation Trust, Chester, CH2 1UL, UK
5. Liverpool University Hospitals NHS Foundation Trust, Mount Vernon Street, L7 8YE, UK

**Corresponding author:**

Mr. Matthew Fok

e. matthew.fok@liverpool.ac.uk

p. Institute of Systems, Molecular and Integrative Biology,

University of Liverpool,

Liverpool,

L69 7BE,

UK

**ORCID ID**: 0000-0003-4091-6569

**Twitter**: @matthewfok

**Supplementary Materials - Index**

Page 3-4

Study Methods

Page 5

Study Limitations

Page 10-11

Page 9

Page 8

| **Supplementary Figures and Tables**  Tablet S1: Baseline characteristics of included studies  Table S2: Risk of bias assessment using Newcastle Ottawa scale (NOS) | Page 6  Page 7 |
| --- | --- |
| Figure S1: Forest plot demonstrating the proportion of seeding rate in patients who underwent biopsy with outliers removed.  Figure S2a: Forest plot demonstrating the proportion of seeding rate in patients who underwent biopsy/biopsy with ablation for primary liver cancer.  Figure S2b: Forest plot demonstrating the proportion of seeding rate in patients who underwent biopsy/biopsy with ablation for secondary liver cancer. (outlier removed)  **References** | Page 9 |
|  |  |

**Search strategy, Data Extraction, Methodological quality Assessment, Statistical Analysis**

The review protocol was registered on the PROSPERO database (CRD42022352550). This systematic review was conducted in accordance with the Preferred Reporting Items for Systematic Reviews and Meta-analyses (PRISMA) statement.

Search strategy

A comprehensive search strategy last updated 11th November 2023 was developed with the search strings “liver neoplasms” AND “seeding” to search the following databases: PubMed, Scopus, and Cochrane. In addition, searches were done on trial registries as well as other grey literature sources. Additional articles were manually screened for using the reference lists of included studies.

Eligibility criteria

Studies with adult patients (>18 y/o) who underwent lesional liver biopsy with or without concurrent ablation were included in this review. Studies published over the last 21 years were included to keep the biopsy and ablation techniques relevant to modern clinical practice. Only studies reported in English were included in this review. Studies with ≤10 patients were excluded. Conference abstracts, systematic reviews, meta-analysis, and case reports were excluded.

*Study screening and selection*

Identified studies were imported into EndNote X9 Referencing Software. A stepwise process was followed to de-duplicate articles in EndNote X9. Following deduplication, the citations and full texts were uploaded onto Rayyan, an online platform which facilitates blinded study screening. Two authors (JEM, SG) conducted the title and abstract screening independently according to the inclusion and exclusion criteria. Following which full text screening was done independently by two authors (JEM, SG). Any discrepancies were resolved with the consensus of the senior review author (RJ).

*Data extraction and measured outcomes*

Data from included studies were extracted by two authors (JEM, SG) onto a predefined spreadsheet in Microsoft Excel. To check for consistency and accuracy the studies were cross checked by the two authors (JEM, SG). Data extracted included baseline demographics, cohort size, tumour details, biopsy type, ablation indication, ablation type, seeding definition, rate of seeding, local recurrence, adverse events, and overall survival. Summary statistics, where available, including counts, percentages, odds-ratio, hazard-ratio, and *p-*values, were extracted.

*Methodological quality assessment of included studies*

Risk of bias was assessed independently by two review authors (SG, MAM) using the Newcastle-Ottawa scale (NOS) and were scored out of 9 points.^(1)^ To check for consistency, the risk of bias results was also reviewed by a third review author (JEM). A breakdown of risk of bias scores is provided in the electronic supplementary material.

Statistical analysis

The metafor statistical package in R (version 4.2.3) was used to perform a random effects meta-analysis of proportions to derive pooled estimates and corresponding 95% confidence interval (CI).^(2)^ The Freeman-Tukey double arcsine transformation was used to stabilise the variances because some of the proportions were close to 0. The percentage of variation attributable to heterogeneity was quantified by the inverse variance index I^2^. Values of 25%, 50%, and 75% were considered as low, moderate, and high heterogeneity respectively. Descriptive statistics for each study were summarized and tabulated. Only papers undergoing biopsy and concurrent ablation within the same session were included within the meta-analysis.

**Study strengths and limitations**

This review has two major strengths: Firstly, this is the first study to directly compare the risk of seeding in patients who underwent biopsy with or without concurrent ablation. Secondly, the study screening process and risk of bias assessment were conducted independently. The limitations of our review are: majority of the papers included are retrospective studies, the relatively small sample size of patients who underwent biopsy with concurrent ablation, including all tumour types in the analysis, the small number of trials assessing secondary liver cancer, and placing an English language restriction to our search strategy. Furthermore, the lack of universally accepted and applied definition of needle track seeding with prospective collection limits the ability of even high-quality meta-analysis to accurately define the frequency and impact of this seemingly rare events.

Table S1: Baseline characteristics of included studies

| **Author** | **Study Design** | **Year** | **Sample Size** | **Tumour type** | **Biopsy/Biopsy with ablation** |
| --- | --- | --- | --- | --- | --- |
| Rodgers et al.^(3)^ | Retrospective | 2003 | 43 | Mets | Biopsy |
| Kosugi et al.^(4)^ | Retrospective | 2003 | 107 | PLC | Biopsy/Biopsy with ablation |
| Ng et al.^(5)^ | Retrospective | 2004 | 91 | PLC | Biopsy |
| Caturelli et al.^(6)^ | Prospective | 2004 | 274 | PLC | Biopsy |
| Chang et al.^(7)^ | Retrospective | 2004 | 1055 | PLC | Biopsy |
| Jones et al.^(8)^ | Retrospective | 2005 | 90 | Mets | Biopsy |
| Livraghi et al.^(9)^ | Retrospective | 2005 | 241 | PLC | Biopsy with ablation |
| Maturen et al.^(10)^ | Retrospective | 2006 | 101 | PLC | Biopsy |
| Colecchia et al.^(11)^ | Retrospective | 2010 | 81 | PLC | Biopsy |
| Ahn et al.^(12)^ | Retrospective | 2011 | 3391 | PLC | Biopsy |
| Matsukuma et al.^(13)^ | Retrospective | 2011 | 181 | PLC | Biopsy |
| Tzortzis et al.^(14)^ | Prospective | 2012 | 134 | PLC & Mets | Biopsy |
| Lopez et al.^(15)^ | Retrospective | 2013 | 23 | PLC | Biopsy with ablation |
| Fuks et al.^(16)^ | Retrospective | 2014 | 75 | PLC | Biopsy |
| Chen et al.^(17)^ | Retrospective | 2015 | 433 | Mets | Biopsy |
| Szpakowski et al.^(18)^ | Retrospective | 2017 | 943 | PLC | Biopsy/Biopsy with ablation |
| Song et al.^(19)^ | Prospective | 2018 | 10 | PLC & Mets | Biopsy with ablation |
| Chen et al.^(20)^ | Retrospective | 2019 | 152 | PLC | Biopsy |
| Schaden et al.^(21)^ | Retrospective | 2020 | 131 | PLC & Mets | Biopsy |
| Fotiadis et al.^(22)^ | Retrospective | 2020 | 741 | Mets | Biopsy |
| Ishikawa et al.^(23)^ | Retrospective | 2021 | 102 | PLC & Mets | Biopsy with ablation |
| Weinfurtner et al.^(24)^ | Prospective | 2021 | 60 | PLC | Biopsy |
| Tse et al.^(25)^ | Retrospective | 2022 | 398 | PLC | Biopsy/Biopsy with ablation |

*PLC – Primary liver cancer *Mets – Metastasis

**Supplementary Material -** Table S2: Risk of bias assessment using Newcastle Ottawa scale (NOS)

|  |  | **SELECTION** |  |  |  | **COMPARABILITY** | **OUTCOME** |  |  |
| --- | --- | --- | --- | --- | --- | --- | --- | --- | --- |
| *Study number* | *Year* | *Representative of the exposed cohort* | *Selection of non exposed cohort* | *Ascertainment of exposure* | *Outcome of interest* | *Comparability of cohorts* | *Assessment of outcomes* | *Sufficient follow-up times* | *Adequacy of follow up* |
| 1 | 2003 | 0 | 1 | 1 | 1 | 0 | 1 | 1 | 1 |
| 2 | 2003 | 0 | 1 | 1 | 1 | 1 | 1 | 1 | 1 |
| 3 | 2004 | 0 | 1 | 0 | 1 | 2 | 1 | 1 | 1 |
| 4 | 2004 | 0 | 1 | 1 | 1 | 1 | 1 | 1 | 1 |
| 5 | 2004 | 0 | 1 | 1 | 1 | 1 | 1 | 0 | 1 |
| 6 | 2005 | 0 | 1 | 0 | 1 | 1 | 1 | 1 | 0 |
| 7 | 2005 | 1 | 1 | 0 | 1 | 1 | 1 | 1 | 1 |
| 8 | 2006 | 0 | 1 | 1 | 1 | 2 | 1 | 1 | 1 |
| 9 | 2010 | 0 | 1 | 1 | 1 | 2 | 0 | 1 | 1 |
| 10 | 2011 | 0 | 1 | 1 | 1 | 2 | 1 | 1 | 1 |
| 11 | 2011 | 0 | 1 | 1 | 1 | 1 | 1 | 0 | 0 |
| 12 | 2012 | 0 | 1 | 1 | 1 | 0 | 1 | 1 | 0 |
| 13 | 2013 | 1 | 1 | 1 | 1 | 1 | 1 | 1 | 1 |
| 14 | 2014 | 1 | 1 | 1 | 1 | 2 | 1 | 1 | 1 |
| 15 | 2015 | 0 | 1 | 1 | 1 | 1 | 1 | 0 | 1 |
| 16 | 2017 | 1 | 1 | 1 | 1 | 2 | 1 | 1 | 1 |
| 17 | 2018 | 0 | 0 | 1 | 1 | 1 | 1 | 0 | 1 |
| 18 | 2019 | 1 | 1 | 1 | 1 | 0 | 1 | 1 | 1 |
| 19 | 2020 | 1 | 1 | 1 | 1 | 2 | 1 | 1 | 1 |
| 20 | 2020 | 1 | 1 | 1 | 1 | 0 | 1 | 1 | 1 |
| 21 | 2021 | 0 | 1 | 1 | 1 | 0 | 1 | 1 | 1 |
| 22 | 2021 | 0 | 1 | 1 | 1 | 2 | 1 | 1 | 1 |
| 23 | 2022 | 1 | 1 | 1 | 1 | 2 | 1 | 1 | 1 |

Figure S1:

Forest plot demonstrating the proportion of seeding rate in patients who underwent biopsy with outliers removed.


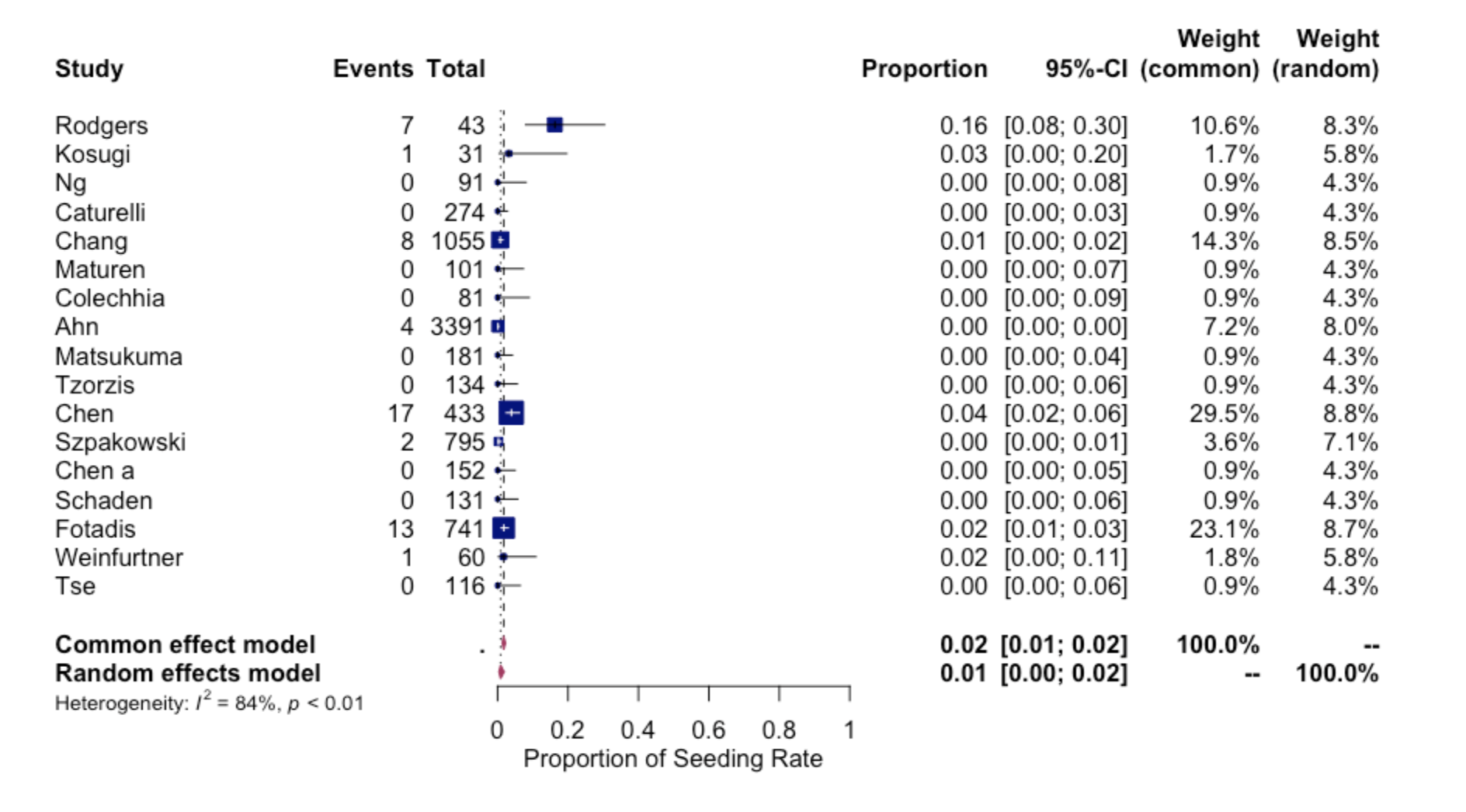


Figure S2a: Forest plot demonstrating the proportion of seeding rate in patients who underwent biopsy/biopsy with ablation for primary liver cancer.


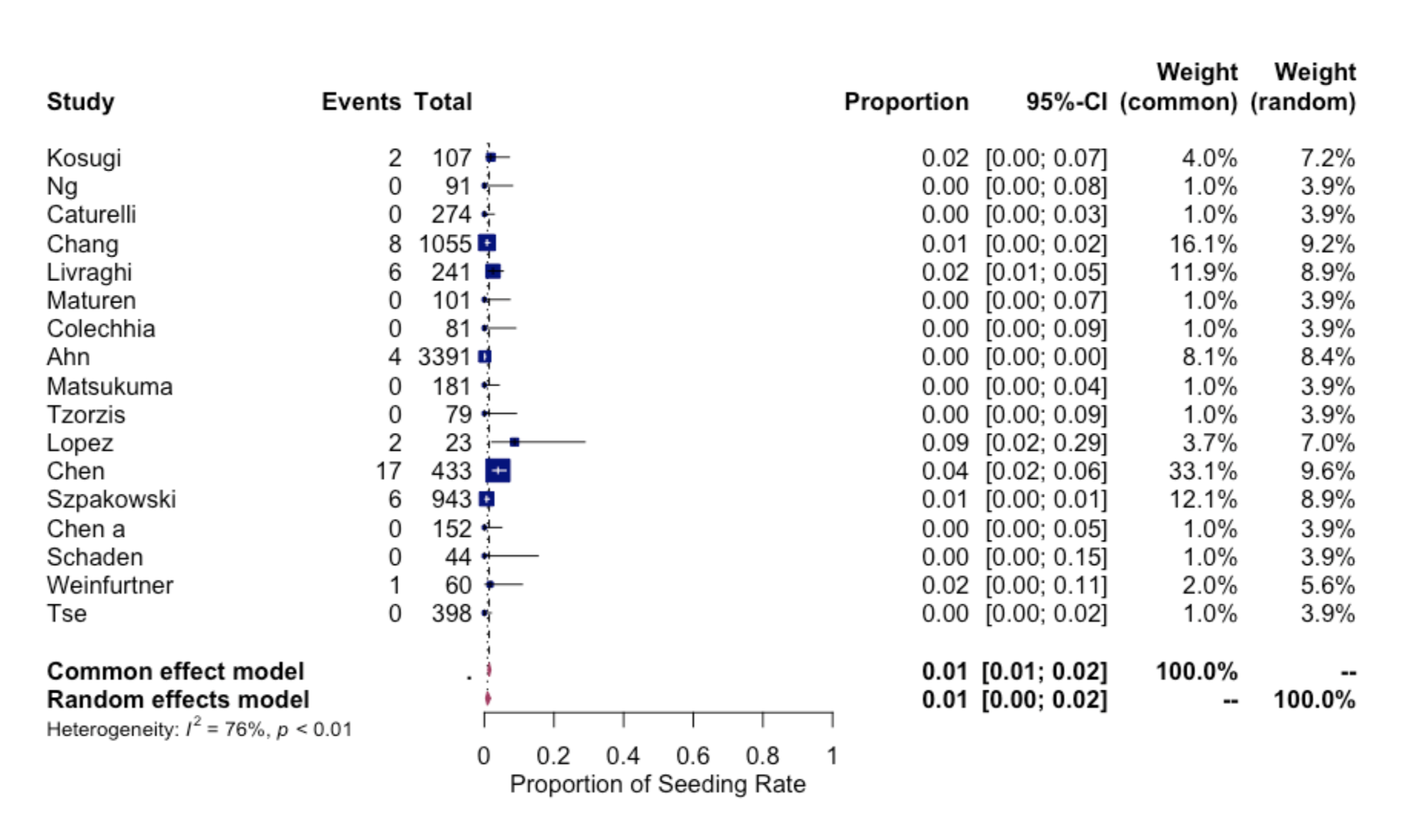


Figure S2b: Forest plot demonstrating the proportion of seeding rate in patients who underwent biopsy/biopsy with ablation for secondary liver cancer. (outlier removed)


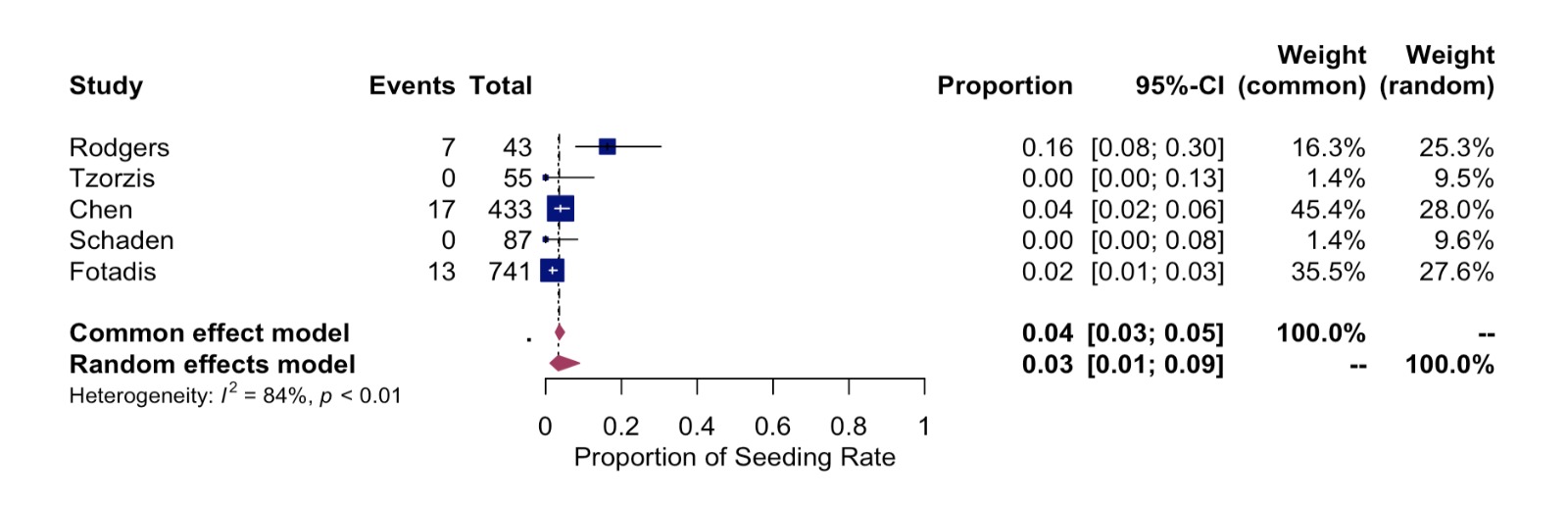


**References:**

1. The Newcastle-Ottawa Scale (NOS) for assessing the quality of nonrandomised studies in meta-analyses.

2. Viechtbauer W. Conducting Meta-Analyses in R with the metafor Package. Journal of Statistical Software. 2010;36(3):1 - 48.

3. Rodgers MS, Collinson R, Desai S, Stubbs RS, McCall JL, Nagorney DM. Risk of dissemination with biopsy of colorectal liver metastases. Diseases of the Colon and Rectum. 2003;46(4):454-8.

4. Kosugi C, Furuse J, Ishii H, Maru Y, Yoshino M, Kinoshita T, et al. Needle Tract Implantation of Hepatocellular Carcinoma and Pancreatic Carcinoma after Ultrasound-guided Percutaneous Puncture: Clinical and Pathologic Characteristics and the Treatment of Needle Tract Implantation. World Journal of Surgery. 2004;28(1):29-32.

5. Ng KKC, Poon RTP, Lo CM, Liu CL, Lam CM, Ng IOL, Fan ST. Impact of Preoperative Fine-needle Aspiration Cytologic Examination on Clinical Outcome in Patients with Hepatocellular Carcinoma in a Tertiary Referral Center. Archives of Surgery. 2004;139(2):193-200.

6. Caturelli E, Solmi L, Anti M, Fusilli S, Roselli P, Andriulli A, et al. Ultrasound guided fine needle biopsy of early hepatocellular carcinoma complicating liver cirrhosis: a multicentre study. Gut. 2004;53(9):1356-62.

7. Chang S, Kim SH, Lim HK, Lee WJ, Choi D, Lim JH. Needle tract implantation after sonographically guided percutaneous biopsy of hepatocellular carcinoma: Evaluation of doubling time, frequency, and features on CT. American Journal of Roentgenology. 2005;185(2):400-5.

8. Jones OM, Rees M, John TG, Bygrave S, Plant G. Biopsy of resectable colorectal liver metastases causes tumour dissemination and adversely affects survival after liver resection. British Journal of Surgery. 2005;92(9):1165-8.

9. Livraghi T, Lazzaroni S, Meloni F, Solbiati L. Risk of tumour seeding after percutaneous radiofrequency ablation for hepatocellular carcinoma. British Journal of Surgery. 2005;92(7):856-8.

10. Maturen KE, Nghiem HV, Marrero JA, Hussain HK, Higgins EG, Fox GA, Francis IR. Lack of tumor seeding of hepatocellular carcinoma after percutaneous needle biopsy using coaxial cutting needle technique. American Journal of Roentgenology. 2006;187(5):1184-7.

11. Colecchia A, Scaioli E, Montrone L, Vestito A, Di Biase AR, Pieri M, et al. Pre-operative liver biopsy in cirrhotic patients with early hepatocellular carcinoma represents a safe and accurate diagnostic tool for tumour grading assessment. Journal of Hepatology. 2011;54(2):300-5.

12. Ahn DW, Shim JH, Yoon JH, Kim CY, Lee HS, Kim YT, Kim YJ. Treatment and clinical outcome of needle-track seeding from hepatocellular carcinoma. The Korean journal of hepatology. 2011;17(2):106-12.

13. Matsukuma S, Sato K. Peritoneal seeding of hepatocellular carcinoma: Clinicopathological characteristics of 17 autopsy cases. Pathology International. 2011;61(6):356-62.

14. Tzortzis D, Revenas K, Deladetsima I, Antoniou E, Tzortzis G. Percutaneous US-guided liver biopsy in focal lesions using a semiautomatic device allowing to perform multiple biopsies in a single-pass. Minerva Gastroenterologica e Dietologica. 2012;58(1):1-8.

15. Lopez KT, Kuwada SK, Wong LL. Consequences of needle tract seeding of hepatocellular cancer after liver transplant. Clinical Transplantation. 2013;27(4):E400-E6.

16. Fuks D, Cauchy F, Fusco G, Paradis V, Durand F, Belghiti J. Preoperative tumour biopsy does not affect the oncologic course of patients with transplantable HCC. Journal of Hepatology. 2014;61(3):589-93.

17. Chen I, Lorentzen T, Linnemann D, Nolsøe CP, Skjoldbye B, Jensen BV, Nielsen D. Seeding after ultrasound-guided percutaneous biopsy of liver metastases in patients with colorectal or breast cancer. Acta Oncologica. 2016;55(5):638-43.

18. Szpakowski JL, Drasin TE, Lyon LL. Rate of seeding with biopsies and ablations of hepatocellular carcinoma: A retrospective cohort study. Hepatol Commun. 2017;1(9):841-51.

19. Song KD, Rhim H, Lee MW, Kang TW, Lim S. A radiofrequency device for tract ablation after liver biopsy: a single-institution human feasibility study. Br J Radiol. 2018;91(1085):20170585.

20. Chen X, Kutaiba N, Ngo B, Goodwin M. Outcome and safety of targeted liver biopsies for indeterminate lesions in patients with chronic liver disease: A single centre experience. Journal of Medical Imaging and Radiation Oncology. 2019;63(2):190-6.

21. Schaffler-Schaden D, Birsak T, Zintl R, Lorber B, Schaffler G. Risk of needle tract seeding after coaxial ultrasound-guided percutaneous biopsy for primary and metastatic tumors of the liver: report of a single institution. Abdominal Radiology. 2020;45(10):3301-6.

22. Fotiadis N, De Paepe KN, Bonne L, Khan N, Riddell A, Turner N, et al. Comparison of a coaxial versus non-coaxial liver biopsy technique in an oncological setting: diagnostic yield, complications and seeding risk. European Radiology. 2020;30(12):6702-8.

23. Ishikawa T, Kodama E, Kobayashi T, Azumi M, Nozawa Y, Iwanaga A, et al. Clinical Efficacy of Liver Tumor Biopsy With Radiofrequency Ablation of the Puncture Route Using a Co-access Needle. Cancer Diagn Progn. 2021;1(5):411-6.

24. Weinfurtner K, Cho J, Ackerman D, Chen JX, Woodard A, Li W, et al. Variability in biopsy quality informs translational research applications in hepatocellular carcinoma. Sci Rep. 2021;11(1):22763.

25. Tse JR, Terashima K, Shen L, McWilliams JP, Lu DSK, Raman SS. Safety of percutaneous, image-guided biopsy of hepatocellular carcinoma with and without concurrent ablation. Abdominal Radiology. 2022;47(8):2640-6.
